# Recognition of outer membrane proteins using multiple feature fusion

**DOI:** 10.3389/fgene.2023.1211020

**Published:** 2023-06-07

**Authors:** Wenxia Su, Xiaojun Qian, Keli Yang, Hui Ding, Chengbing Huang, Zhaoyue Zhang

**Affiliations:** ^1^ College of Science, Inner Mongolia Agriculture University, Hohhot, China; ^2^ School of Life Science and Technology, Center for Information Biology, University of Electronic Science and Technology of China, Chengdu, China; ^3^ Nonlinear Research Institute, Baoji University of Arts and Sciences, Baoji, China; ^4^ School of Computer Science and Technology, Aba Teachers University, Aba, China; ^5^ School of Healthcare Technology, Chengdu Neusoft University, Chengdu, China

**Keywords:** outer membrane protein, pseudo amino acid composition, support vector machine, jackknife test, prediction model

## Abstract

**Introduction:** Outer membrane proteins are crucial in maintaining the structural stability and permeability of the outer membrane. Outer membrane proteins exhibit several functions such as antigenicity and strong immunogenicity, which have potential applications in clinical diagnosis and disease prevention. However, wet experiments for studying OMPs are time and capital-intensive, thereby necessitating the use of computational methods for their identification.

**Methods:** In this study, we developed a computational model to predict outer membrane proteins. The non-redundant dataset consists of a positive set of 208 outer membrane proteins and a negative set of 876 non-outer membrane proteins. In this study, we employed the pseudo amino acid composition method to extract feature vectors and subsequently utilized the support vector machine for prediction.

**Results and Discussion:** In the Jackknife cross-validation, the overall accuracy and the area under receiver operating characteristic curve were observed to be 93.19% and 0.966, respectively. These results demonstrate that our model can produce accurate predictions, and could serve as a valuable guide for experimental research on outer membrane proteins.

## 1 Introduction

Outer membrane proteins (OMPs) are a special type of proteins that are found in the outermost membranes of Gram-negative bacteria, mitochondria, and chloroplasts (Rollauer et al., 2015; Qi et al., 2022). OMPs serve a wide range of functions, including acting as adhesion factors in virulence, channels for small hydrophilic molecules, enzymes in biochemical reactions, and antigens in immune responses. They also work in concert with other substances to enhance the bacteria pathogenicity. Recent research on OMPs has revealed their potential for clinical diagnosis and disease prevention. Several published studies have explored OMPs as potential vaccine candidates (Budiardjo et al., 2021; Fahie et al., 2021; Cheng et al., 2022; Yu et al., 2022). The functions are determined by the OMP’s structure and the way it interacts with other molecules. OMPs are typically composed of a transmembrane β-barrel architecture, providing permeability to the outer membrane and maintaining structural stability. Among different types of OMPs, β-buckets consist of varying even numbers of β-folding sheets, ranging from 8 to 26 (Rollauer et al., 2015). The specific composition of the β-barrel architecture is determined by the amino acid sequence of the OMPs. Mutations in sequences can impact the stability and function of the protein.

Distinguishing OMPs from non-OMPs can aid researchers in identifying promising vaccine targets, developing new antibiotics and therapeutics, and understanding the evolution of Gram-negative bacteria. Despite their distinctive β-barrel structure, OMPs are exposed to numerous charged and polar residues in the membrane, making it challenging to distinguish them from non-OMPs. This is a primary challenge and a significant obstacle in the research process, given the considerable time and capital costs associated with laboratory studies of OMPs. As a result, OMP prediction has tremendous significance for the scientific community. Currently, various machine learning methods have been used for the identification of OMPs, such as support vector machine (SVM) (Park et al., 2005; Gromiha et al., 2006; Hu et al., 2017; Zhang et al., 2021), k-nearest neighbor (K-NN) method (Yan et al., 2008), neural networks (NN) (Hu et al., 2017). These methods utilize the amino acid composition, and physical and chemical properties of the amino acid sequences to construct the prediction models. Gromiha and Suwa (2003); Gromiha and Suwa (2005) developed multiple OMP prediction methods based on amino acid composition, residue pair preference, and motif sequence. However, these methods only achieved prediction accuracies of 80%–90%. Subsequently, a machine learning algorithm was proposed with a higher accuracy ranging from 90% to 94% (Gromiha et al., 2005; Gromiha et al., 2006). Lin (2008) further improved the OMP prediction model by introducing the Incremental Diversity with Quality Distinctness analysis, which combines the Markov discriminant method and the pseudo amino acid composition (Pse-AAC). Despite the progress made in OMP predictions, there is still room for further improvement in prediction quality.

In this article, we proposed a novel method for predicting OMPs that combines Pse-AAC and SVM. To extract the features for amino acid composition and physical and chemical characteristics of amino acids, we used the Pse-AAC feature extraction method. Additionally, we introduced multi-level amino acid residue index correlation coefficients such as hydrophobic value, average polarity, and solvation-free energy to enhance the accuracy of our prediction model. To assess the effectiveness and reliability of our approach, we also conducted a comprehensive comparison and analysis of our proposed model with existing methods for predicting OMPs. Our developed approach will be useful for distinguishing OMPs from non-OMPs.

## 2 Materials and methods

### 2.1 Datasets

The construction of a reliable dataset is the basis for developing an accurate outer membrane protein prediction model (Su et al., 2021). A well-designed dataset is crucial for developing effective algorithms and an objective evaluation and prediction system. In this paper, membrane proteins were extracted from the PSORT-B database (https://www.psort.org/) (Gardy et al., 2003), and globular proteins were extracted from the PDB40D of SCOP_1.37 database (http://scop.mrc-lmb.cam.ac.uk/scop/) (Andreeva et al., 2020). As a result, a total of 208 OMPs were selected as the positive set, while 879 non-OMPs were chosen as the negative set. The negative set included 206 inner membrane proteins and 673 globular proteins. The globular protein dataset contained 154 complete α proteins, 156 complete β proteins, 184 α+β proteins, and 179 α/β proteins. Since the sequence homology of each protein class was less than 40%, proteins in each database were not similar and were de-redundant.

### 2.2 Feature encoding

To construct a prediction model, it is necessary to represent the protein sequences as mathematical vectors. This conversion is commonly known as feature extraction (Basith et al., 2020; Dao et al., 2022b; Zhang Z.-Y. et al., 2022; Hunt et al., 2022; Karuna Nidhi et al., 2022; Sun et al., 2022; Tran and Nguyen, 2022; Wang et al., 2022; Yang et al., 2022). The amino acid composition (ACC) of the protein has a great impact on protein classification research (Awais et al., 2021; Shoombuatong et al., 2022b; Manavalan and Patra, 2022; Rout et al., 2022; Zhu et al., 2022). By using the ACC, a protein sequence can be represented as a 20-D (dimension) vector as follows:

$$V_{AAC}\left(S\right)={\left(v_{1},v_{2},v_{3},\cdots ,v_{20}\right)}^{T}$$ (1)

In Eq. 1, $v_{i}=f_{i}/\sum f_{i}$ , $f_{i}$ represented the number of the $i\;\left(i=1,2,\cdots ,20\right)$ amino acid in the protein sequence.

The type of amino acids is determined by their side chains, as the 20 types of amino acid side chains differ in shape, size, negativity, hydrophobicity, and acid-base properties. The distinct characteristics of the 20 amino acid side chains result in various combinations of amino acid sequences that exhibit different structures and functions. Therefore, algorithms based on the physicochemical properties of amino acids are another major category of feature extraction methods. Pse-AAC, originally proposed by Chou, is a feature extraction algorithm, that is, based on the physical and chemical properties of amino acids (Chou, 2005). By using Pse-AAC, a protein sample can be represented as follows:

$${V_{PAAC}=\left[x_{1},\ldots ,x_{20},x_{20+1},\ldots ,x_{20+\lambda }\right]}^{T}$$ (2)

where the first 20 numbers in Eq. 2 are the classic AAC features, and the next λ discrete numbers represent the position information of residues in amino acid sequences. For different problems, the optimal value of λ may vary. In this study, we selected the optimal value of λ that yielded the highest sensitivity through the jackknife test.

### 2.3 Support vector machine

SVM is a powerful supervised machine learning classification method based on statistical learning theory (Manavalan et al., 2019). It was originally designed based on the idea of the generalized linear classifier. First, features were mapped to high-dimensional space. Next, a separating hyperplane is constructed to separate the two categories in the high-dimensional feature space (Vapnik and Control, 2019). To avoid expensive computations, the mapping function only involves the relatively low-dimensional vector in the input space and the dot product in the feature space. The global optimization approach and avoidance of overfitting in SVM have made it a successful tool for addressing various bioinformatics problems (Zhang H. et al., 2022). In this paper, the support vector machine (SVM) was implemented using the widely used software LIBSVM (http://www.csie.ntu.edu.tw/∼cjlin/libsvm) (Chang and Lin, 2011). The radial basis function which is defined as $K\left(x_{i},x_{j}\right)=\exp\left(-\gamma {\left\|x_{i}-x_{j}\right\|}^{2}\right)$ was chosen as the kernel function. The regularization parameter *C* and the kernel width parameter *γ* were optimized on the training set using a grid search strategy.

### 2.4 Evaluation methods

At present, k-fold cross-validation and jackknife cross-validation are widely used for prediction evaluation (Tabaie et al., 2021; Dao et al., 2022a; Xiao et al., 2022; Zhou et al., 2022). The jackknife test is a type of cross-validation that involves leaving one observation out of the dataset at a time and using the remaining observations to train a model. This process is repeated for each observation in the dataset, resulting in n different models, where n is the number of observations in the dataset. In this article, we used the Jackknife test to evaluate the prediction results. The sensitivity (*S*_*n*_), specificity (*S*_*p*_), average accuracy (*AA*), overall prediction accuracy (*OA*), and Matthew’s correlation coefficient (*MCC*), the area under ROC curve (auROC) were used to evaluate the prediction performance of the algorithm (Yang et al., 2021; Zhang Q. et al., 2022). The evaluation metrics are defined as follows:

$$Sn=\frac{TP}{TP+FN}$$ (3)

$$Sp=\frac{TN}{TN+FP}$$ (4)

$$MCC=\frac{TP\times TN-FP\times FN}{\sqrt{\left(TP+FN\right)\left(TP+FP\right)\left(TN+FP\right)\left(TN+FN\right)}}$$ (5)

$$OA=\frac{TP+TN}{TP+TN+FN+FP}$$ (6)

$$AA=\frac{1}{2}\times \left(\frac{TP}{TP+FN}+\frac{TN}{TN+FP}\right)$$ (7)

where *TP* represents the number of the positive sample correctly identified, *FN* represents the positive sample wrongly identified as a negative sample, *FP* represents the negative sample wrongly identified as a positive sample, and *TN* represents the negative sample correctly identified. AuROC is an indicator that relates to the receiver operating characteristic (ROC) curve, which is a plot of a series of continuous (1-*Sp*) values on the horizontal axis against their corresponding Sn values on the vertical axis. The ROC curve is a useful tool for evaluating the sensitivity and specificity of a model (Hasan et al., 2022; Jeon et al., 2022). AuROC is calculated in this study as an indicator of classification ability and performance. A larger auROC value indicates better performance and classification ability of the model.

## 3 Results and discussion

### 3.1 Model performance

In this study, the proteins were first obtained in FASTA format and then the PseAAC program (Shen and Chou, 2008) was used to extract the feature vectors of pseudo amino acid components. To achieve relatively optimal prediction results, different parameters were selected to extract pseudo amino acid component feature vectors of protein sequences. Specifically, feature vectors were extracted using different values of ω (the weight factor) including 0.1, 0.2, 0.3, 0.4, 0.5, and 0.6, and λ was taken as either 3 or 5. The extracted feature vectors were then used for prediction using different values of γ including 0.04, 0.05, 0.06, 0.07, 0.08, and 0.09. The SVM model was trained using svm-train in LIBSVM, and the optimal parameter array and optimal feature subset were searched from the prediction results. Only the γ value that achieved the optimal prediction result was selected and listed in Table 1.

**TABLE 1:** The performance comparison of prediction models under different parameter conditions.

ω , λ , γ	Sn(%)	*Sp*(%)	*MCC*(%)	*OA* (%)	*AA* (%)	AuROC
0.1,3,0.05	78.37	96.59	77.16	93.10	87.48	0.962
0.2,3,0.08	78.85	96.59	77.49	93.19	87.72	0.966
0.3,3,0.09	75.96	96.59	75.46	92.64	86.27	0.965
0.4,3,0.08	79.33	95.79	75.97	92.64	87.56	0.962
0.5,3,0.09	79.33	95.79	75.97	92.64	87.56	0.961
0.6,3,0.09	79.81	95.90	76.57	92.82	87.86	0.958
0.1,5,0.07	79.81	96.59	78.17	93.38	88.20	0.965
0.2,5,0.09	79.81	95.56	75.79	92.55	87.69	0.968
0.3,5,0.09	80.77	95.45	76.22	92.64	88.11	0.966
0.4,5,0.08	81.73	95.11	76.15	92.55	88.42	0.964
0.5,5,0.07	84.61	95.11	78.19	93.10	89.86	0.963
0.6,5,0.07	81.25	94.43	74.34	91.90	87.84	0.956

In this study, the benchmark dataset consisted of 208 OMPs and 879 non-OMPs. Due to this imbalanced dataset, using average accuracy as the sole evaluation criterion may lead to skewed results toward the negative sets. Thus, the paper used overall accuracy as the main criterion for model evaluation. By analyzing the data in Table 1, it was observed that high prediction sensitivity was achieved using Jackknife cross-validation with different parameters. And, with the best prediction result obtained with a weight factor of 0.5, the parameter λ taking 5, γ taking 0.07, resulting in an overall accuracy of 93.10%.

### 3.2 Model comparison

Various methods have been proposed by different researchers to predict and distinguish OMPs from other types of membrane proteins. Wu et al. (2007) proposed a prediction method that uses information differences to compare the distribution of subsequences and residual sequences, resulting in a prediction accuracy of 99.20%. Yan et al. (2008) proposed a method based on the K-nearest neighbor (KNN) method, which predicted the weighted Euclidean distance calculated by residual synthesis and achieved a recognition accuracy of 96.1%, sensitivity of 87.5%, specificity of 98.2% with 0.873 MCC. Gromiha et al. (2006) discriminate of OMPs and non-OMPs using different machine learning approaches, the best performance achieved sensitivity of 84.6%, specificity of 95.8% and accuracy of 93.7%. And the SVM-based model achieved sensitivity of 72.6%, specificity of 98.2% and accuracy of 93.3%. Park et al. (2005) proposed an SVM method that considers both amino acid composition and residue pair information, achieving sensitivity of 90.9 %, specificity of 94.7%, MCC 0.816 of and accuracy of 93.9%. Gao et al. (2010) developed a method that combined the structural and physicochemical characteristics of sequence-derived proteins with amino acid composition to distinguish OMPs and non-OMPs using SVM, with an overall accuracy of 97.8%, sensitivity of 91.8 %, specificity of 99.2% and MCC 0.928.

In this paper, the model constructed using the SVM algorithm achieved an overall accuracy of 93.10% and auROC of 0.963 under Jackknife cross-validation, respectively. Besides, the sensitivity, specificity, MCC, and average accuracy were found to be 84.61%, 95.11%, 78.19%, and 89.86%, respectively. Compared to previous SVM-based models, some progress has been made.

## 4 Conclusion

This article focused on the prediction and recognition of OMPs using the method of combining Pse-AAC with SVM. The study achieved good results with the Pse-AAC method, which not only considers the content of 20 natural amino acids in each protein sequence but also includes the correlation between various amino acids, such as physical and chemical properties. This approach is more advanced than traditional methods that only consider amino acid composition, leading to more accurate prediction results. SVM is a widely used algorithm in bioinformatics (Hasan et al., 2020; Shoombuatong et al., 2022a; Bupi et al., 2023), and applying it to the prediction of OMPs is an inevitable trend in current research. The constructed model using the SVM algorithm achieved high performance with an overall accuracy of 93.10% and auROC of 0.963 under Jackknife cross-validation. The sensitivity, specificity, Matthew correlation coefficient, and average accuracy achieved 84.61%, 95.11%, 78.19%, and 89.86%, respectively. However, while feature extraction algorithms have been widely used in prediction methods and have achieved good performance, the relationship between the extracted information and protein structure and function needs to be further explored. This challenge will undoubtedly be the focus of our future research efforts aimed at identifying OMPs. The development of accurate prediction models for OMPs has the potential to significantly impact fields ranging from antibiotic discovery and vaccine development to biotechnology and bacterial diagnostics.

## Data Availability

The original contributions presented in the study are included in the article/Supplementary Material, further inquiries can be directed to the corresponding authors.
